# Improvement of Physical and Electrical Characteristics in 4H-SiC MOS Capacitors Using AlON Thin Films Fabricated via Plasma-Enhanced Atomic Layer Deposition

**DOI:** 10.3390/ma18194531

**Published:** 2025-09-29

**Authors:** Zhaopeng Bai, Chengxi Ding, Yunduo Guo, Man Luo, Zimo Zhou, Lin Gu, Qingchun Zhang, Hongping Ma

**Affiliations:** 1 Institute of Wide Bandgap Semiconductors and Future Lighting, College of Intelligent Robotics and Advanced Manufacturing, Fudan University, Shanghai 200433, China; zpbai24@m.fudan.edu.cn (Z.B.); 23210860003@m.fudan.edu.cn (C.D.); 22210860001@m.fudan.edu.cn (Y.G.); 24210720123@m.fudan.edu.cn (M.L.); 24210860099@m.fudan.edu.cn (Z.Z.); 24110860041@m.fudan.edu.cn (L.G.); qingchun_zhang@fudan.edu.cn (Q.Z.); 2 Shanghai Research Center for Silicon Carbide Power Devices Engineering & Technology, Fudan University, Shanghai 200433, China; 3 Institute of Wide Bandgap Semiconductor Materials and Devices, Research Institute of Fudan University in Ningbo, Ningbo 315327, China

**Keywords:** SiC MOS, AlON, PEALD, interfaces, electrical properties

## Abstract

In this study, we investigate the improvement of physical and electrical characteristics in 4H-silicon carbide (SiC) MOS capacitors using Aluminum Oxynitride (AlON) thin films fabricated via Plasma-Enhanced Atomic Layer Deposition (PEALD). AlON thin films are grown on SiC substrates using a high ratio of NH_3_ and O_2_ as nitrogen and oxygen sources through PEALD technology, with improved material properties and electrical performance. The AlON films exhibited excellent thickness uniformity, with a minimal error of only 0.14%, a high refractive index of 1.90, and a low surface roughness of 0.912 nm, demonstrating the precision of the PEALD process. Through XPS depth profiling and electrical characterization, it was found that the AlON/SiC interface showed a smooth transition from Al-N and Al-O at the surface to Al-O-Si at the interface, ensuring robust bonding. Electrical measurements indicated that the SiC/AlON MOS capacitors demonstrated Type I band alignment with a valence band offset of 1.68 eV and a conduction band offset of 1.16 eV. Additionally, the device demonstrated a low interface state density (D_it_) of 7.6 × 10^11^ cm^−2^·eV^−1^ with a high breakdown field strength of 10.4 MV/cm. The results highlight AlON’s potential for enhancing the performance of high-voltage, high-power SiC devices.

## 1. Introduction

Compared to other wide bandgap semiconductor materials, silicon carbide (SiC) offers a unique advantage in that it can directly generate a SiO_2_ dielectric layer through thermal oxidation, providing inherent benefits in the fabrication of power MOSFETs. However, unlike silicon (Si), 4H-SiC contains a significant amount of carbon, which makes the thermal oxidation process for growing SiO_2_ on SiC different from that on Si. The interface between SiC and SiO_2_ exhibits greater complexity compared to the Si/SiO_2_ interface, with a significantly higher density of interface states. These states have a profound impact on carrier mobility at the surface, resulting in much lower surface mobility compared to the bulk, thereby causing considerable degradation in device performance [1,2,3]. According to Gauss’s law of $E_{ins}=\frac{\kappa_{s}}{\kappa_{ins}}E_{s}$ [4,5], the dielectric constant of 4H-SiC exceeds that of SiO_2_ by a factor of 2.5. This discrepancy results in the electric field intensity in the SiO_2_ gate oxide layer being 2.5 times greater than that in the SiC, which accelerates the breakdown of the SiO_2_ layer. Devices with the SiO_2_/4H-SiC interface will exhibit a lower critical breakdown field strength compared to 4H-SiC devices. The lower dielectric constant of SiO_2_ thus hampers the full utilization of 4H-SiC’s high critical breakdown field strength properties [6].

Under the same thickness and capacitance conditions, high-k gate dielectric materials exhibit a physically effective thickness greater than that of SiO_2_, leading to improvements in gate leakage current and breakdown field strength. Additionally, the density of interface states at the high-k/4H-SiC interface is smaller compared to that at the SiO_2_/4H-SiC interface, resulting in a significant reduction in parasitic leakage currents and improving the overall stability of the device [7].

HfO_2_ and ZrO_2_ are well-known high-k dielectric materials and have been successfully integrated into commercial silicon device processing technologies. However, using HfO_2_ and ZrO_2_ as dielectric materials on 4H-SiC presents a challenge due to the relatively low barrier height between HfO_2_/ZrO_2_ and 4H-SiC. This results in electron injection from the conduction band of 4H-SiC into the dielectric even at very low electric fields, leading to significant leakage currents [8].

Among high-k materials, Aluminum Oxide (Al_2_O_3_) has garnered significant interest due to its exceptional electrical characteristics and minimal leakage current. In the context of SiC MOS devices, Al_2_O_3_ films have shown remarkable performance, exhibiting low leakage current and a reduced interface state density (D_it_), making them an ideal choice for gate dielectric applications [9,10]. These characteristics make Al_2_O_3_ an ideal choice for enhancing the performance of SiC MOSFETs. However, there are certain drawbacks associated with Al_2_O_3_ gate dielectrics in applications, particularly in terms of the flatband voltage (V_FB_) shift. This shift is primarily caused by interface-related issues between the Al_2_O_3_ dielectric and the SiC substrate. At this interface, defects and charge trapping states are likely present, which contribute to the observed voltage shift. These imperfections can cause shifts in the electrical performance of the gate dielectric, thereby limiting its widespread use in high-reliability applications [11,12]. The SiC/Aluminum Nitride (AlN) MOS structure has been proposed as a viable alternative, exhibiting improved breakdown voltage characteristics and a reduction in V_FB_ shift. However, due to its relatively low barrier height, this structure exhibits higher leakage current, which compromises the stability of the device [13,14,15]. By incorporating the Al_2_O_3_ and AlN in specific proportions to form Aluminum Oxynitride (AlON), the advantages of both Al_2_O_3_ and AlN can be effectively combined, resulting in a high-performance gate dielectric material [16,17,18].

AlON gate dielectrics have been extensively studied, and notable progress has been made in enhancing material properties. In traditional methods, N_2_ and H_2_O are used as nitrogen and oxygen sources for growing AlON thin films on SiC substrates. However, this approach results in films with a high interface state density, approximately 8.93 × 10^12^ cm^−2^ eV^−1^, which limits their application in devices [13]. To overcome this issue, research using NH_3_ as the nitrogen source has shown that NH_3_ exhibits stronger reactivity with the aluminum precursor Trimethylaluminum (TMA) in PEALD processes, effectively promoting AlON growth and improving film quality [19]. Furthermore, studies employing PEALD with N_2_ and O_2_ as nitrogen and oxygen sources for growing AlON layers on SiC substrates have revealed a complex relationship between nitrogen content, defect density, leakage current, and other electrical properties [16]. Further investigation suggests that by employing a low-temperature PEALD process (185 °C) with a high ratio of NH_3_ and O_2_ (>85%), not only is a uniform nitrogen doping distribution achieved, but AlON films also exhibit a notable decrease in surface roughness. This results in superior performance for applications in gate dielectrics and other high-performance applications [18]. However, this study was conducted on Si substrates and focused primarily on material properties without investigating electrical characteristics. Therefore, AlON thin films are grown on SiC substrates using a high ratio of NH_3_ and O_2_ as nitrogen and oxygen sources through PEALD technology firstly, with a comprehensive evaluation of both material properties and electrical performance. This can offer new theoretical foundations for the further enhancement and optimization of SiC MOS devices.

## 2. Experimental Methods

### 2.1. Fabrication

**Figure 1:**
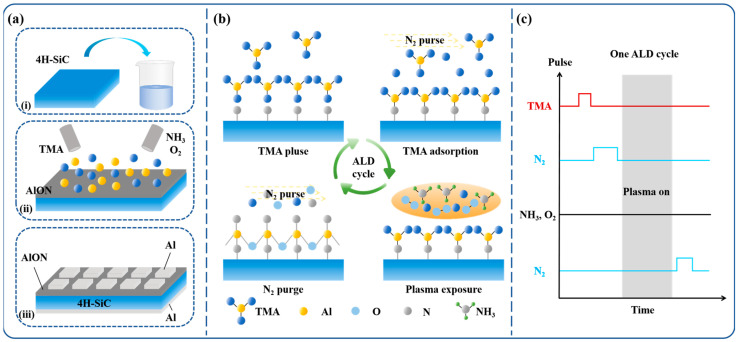
(**a**) Experimental procedure. (**b**) ALD cycle process. (**c**) ALD parameters used for AlON film growth [15].

The procedure for sample preparation is shown in Figure 1a. N-type 4H-SiC epitaxial wafers with a 4° off-cut angle along the (0001) plane were chosen as the substrates for this study. The epitaxial layer had a doping concentration of 8.5 × 10^15^ cm^−3^ and a thickness of 10 μm. As shown in Figure 1a, the fabrication process of AlON/SiC MOS is presented. It consists of three steps: (i) The substrate surface was first treated with a buffered oxide etch (BOE) solution to eliminate native oxides. Following this, the surface was further purified by rinsing with acetone and isopropyl alcohol to remove any organic contaminants. (ii) The cleaned substrate was then transferred to the reaction chamber of the ALD system. TMA was employed as the aluminum precursor, and a mixture of NH_3_ and O_2_ gases, in a 95:5 ratio, served as the nitrogen and oxygen precursors, respectively. (iii) The growth of Al electrodes was carried out separately on the front and back sides.

The specific deposition process is depicted in Figure 1b. TMA was introduced into the reaction chamber, where it adsorbs onto the substrate surface. Subsequently, N_2_ pulses were introduced to remove any remaining precursor. NH_3_ was continuously supplied to the SiC wafer throughout the deposition, with a constant flow rate of 100 sccm. A radio frequency (RF) generator operating at 200 W was then used to initiate the plasma process, triggering a self-limiting reaction between NH_3_ plasma and TMA. Afterward, an additional N_2_ purge was conducted to remove any remaining byproducts.

The parameters for the ALD process used in AlON deposition are shown in Figure 1c. Each cycle of ALD consists of four distinct stages: (i) a 1 s TMA pulse, (ii) a 5 s N_2_ purge, (iii) a 10 s plasma activation, and (iv) a concluding 5 s N_2_ purge. This process was repeated 400 times, resulting in the deposition of an approximately 40 nm thick AlON film on the substrate. Following the AlON film deposition, a square array of Al electrodes, each 200 μm × 200 μm in size and 200 nm thick, was deposited on the front surface using magnetron sputtering. A 200 nm thick metal Al layer was then applied to the back surface to function as the back electrode.

### 2.2. Characterization

The AlON thin films and AlON/SiC interfaces were comprehensively evaluated using a variety of physical, chemical, and electrical characterization techniques. Ellipsometry was used to measure the thickness and optical properties of the AlON films, with a 70° incident angle and a wavelength range from 190 nm to 900 nm (HORIBA, Longjumeau, France SAS). Surface roughness and morphological analysis were conducted using atomic force microscopy (AFM, BRUKER BRK0003, Ettlingen, Germany), employing a scan area of 5 × 5 μm^2^. The crystalline structure and film quality were assessed using X-ray diffraction (XRD) with a Bruker D8 Advance diffractometer (BRUKER, Ettlingen, Germany), employing Cu Kα radiation (40 kV, 40 mA, λ = 1.54 Å). Surface chemical states were analyzed using X-ray photoelectron spectroscopy (XPS, Thermo Scientific ESCALAB 250Xi, Waltham, MA, USA) with a monochromatic Al Kα X-ray source (1486.6 eV, 150 W). The spectra were calibrated with reference to the C 1s peak at 284.8 eV.

The electrical properties of the AlON/SiC MOS capacitors were evaluated using a TS2000-HP probe station (MPI Corporation, Hsinchu, Taiwan) and a Keithley 4200A-S semiconductor parameter analyzer (Tektronix Inc., Beaverton, OR, USA). To assess the interface state density (D_it_), capacitance–voltage (C-V) measurements were carried out at both high and low frequencies, providing critical insights into the device characteristics. The shift in flatband voltage was utilized to estimate the effective fixed charge density (N_eff_). High-frequency hysteresis C-V tests were carried out to investigate the near-interface traps (N_ITs_). Additionally, current–voltage (I-V) measurements were taken to analyze the device behavior under varying applied voltages, and Fowler–Nordheim (FN) tunneling analysis was performed to extract the barrier height between AlON and SiC.

## 3. Results and Discussion

### 3.1. Quality of AlON Films

**Figure 2:**
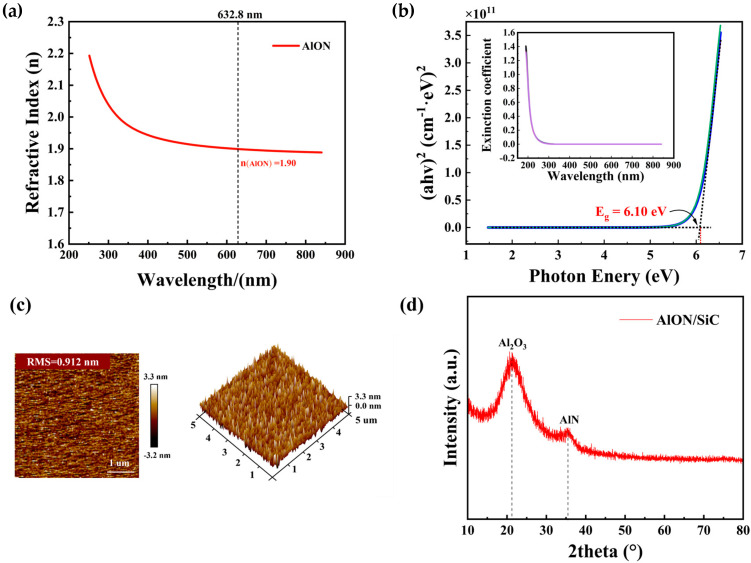
(**a**) SE results of SiC/AlON samples for fitted refractive index. (**b**) Tauc plots, with an inset displaying the extinction coefficient as a function of wavelength. (**c**) 2D, 3D AFM images of AlON film, with scanned areas of 5 × 5 µm^2^. (**d**) XRD of AlON films deposited onto 4H-SiC.

The quality of the AlON thin films grown on SiC substrates through PEALD was assessed using SE, AFM, and XRD. These techniques were applied to systematically examine the films’ optical characteristics, surface morphology, and crystalline structure. The AlON film thickness and refractive index were obtained from SE measurements, with the refractive index fitted using the Cauchy dispersion model, as shown in Figure 2a [20]. The measured refractive index of AlON at a wavelength of 632.8 nm was found to be 1.90, falling between the values of Al_2_O_3_ (1.65) [10] and AlN (1.95) [15]. This finding aligns with previous research, which demonstrated a steady increase in the refractive index of AlON films with an elevated NH_3_:O_2_ ratio [21], further confirming the impact of nitrogen doping on the optical properties.

The refractive index serves as an indicator of a film’s structural properties. The lower refractive index is indicative of a more porous structure or the presence of impurities, whereas a higher refractive index typically signifies a denser, more compact film structure [22,23]. To assess thickness uniformity, SE measurements were performed at five distinct locations on the AlON thin film. The fitted thickness average value was 45.8 nm, with a standard deviation of 0.066 nm, yielding a relative error of only 0.14%. These results indicate a high degree of thickness uniformity for the AlON film deposited by PEALD.

The optical bandgap (E_g_) of the AlON films was determined by constructing a Tauc plot, which correlates the absorption coefficient (α) to photon energy (hv) using the equation below [24]:

(1) $$\left(\alpha hv{)}^{n}\propto (hv-E_{g}\right)$$

where h denotes the Planck constant, v refers to the photon frequency and α presents the absorption value. As AlON is a direct bandgap material and n = 2 is justified [25]. The absorption value was obtained by applying the following equation [26,27]:

(2) $$\alpha =\frac{4\pi k}{\lambda }$$

where k represents the extinction coefficient, and λ is the wavelength of the incident light. By utilizing this coefficient, the absorption coefficient can be derived, which in turn allows for the calculation of the optical bandgap of the AlON films. Through these calculations, the optical bandgap was determined to be 6.10 eV. This value was obtained by applying Tauc’s equation to correlate photon energy (hv) with absorption, followed by a linear extrapolation of the resulting plot, as shown in Figure 2b.

AFM was employed to investigate the surface morphology of the MOS capacitor, with a scan area of 5 × 5 μm^2^. The AFM topographical image is shown in Figure 2c. The root mean square (RMS) roughness of the gate dielectric was measured to be 0.912 nm, indicating a highly smooth surface. This low roughness highlights the effectiveness of ALD in producing high-quality AlON films with superior surface characteristics.

The XRD analysis of the AlON film revealed relatively broad diffraction peaks with low intensity, indicating its low crystallinity. Two minor peaks were observed at approximately 2θ = 21.6° and 35.2°. Based on the standard diffraction peak positions and characteristics of Al_2_O_3_ and AlN phases [28,29], it is inferred that trace amounts of crystalline Al_2_O_3_ and AlN phases are present within the AlON film. Importantly, no additional diffraction peaks were detected, indicating that the AlON film is primarily composed of an amorphous phase or contains nanocrystalline regions. This structural characteristic can be attributed to the controlled parameters of the ALD process, which favors the amorphization of the material. In this study, a 200 °C deposition temperature and a 10 s NH_3_/O_2_ plasma pulse proved inadequate to promote surface atom mobility or initiate nucleation, leading to the formation of an amorphous AlON film. The amorphous nature of the film is advantageous for gate dielectric applications, as it helps suppress carrier tunneling [18].

### 3.2. AlON/SiC Interface Properties

**Figure 3:**
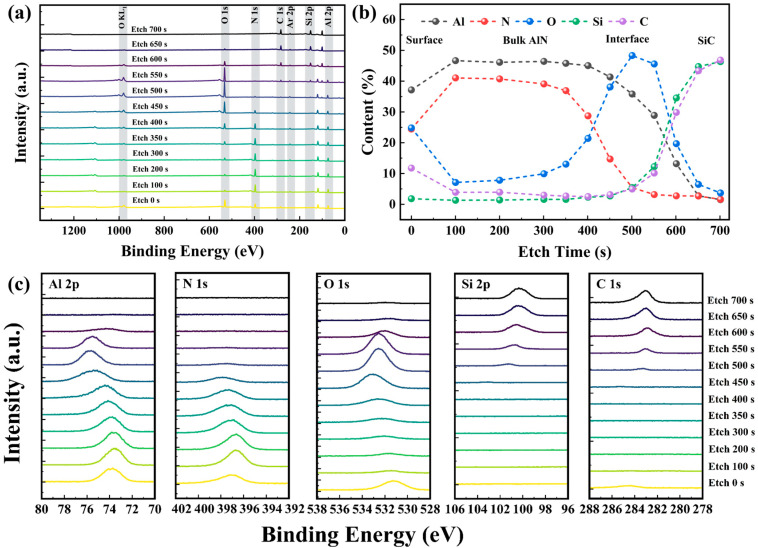
(**a**) XPS survey spectra acquired at various etching times. (**b**) Atomic concentrations of different elements of various etching times. (**c**) High-resolution XPS spectra of Al 2p, N 1s, O 1s, Si 2p, and C 1s for various etching times.

The elemental composition and chemical states of the AlON films deposited on 4H-SiC via PEALD were examined using XPS depth profiling, with varied etching times. Initially, survey scans, high-resolution spectra, and valence band spectra were obtained from the surface of the film to investigate its chemical composition and bonding properties. Subsequently, multiple cycles of Ar^+^ ion etching were performed with etching time of 100, 200, 300, 350, 400, 450, 500, 550, 600, 650, and 700 s. These different etching times correspond to distinct sampling depths within the sample. Following each etching step, the corresponding XPS spectra were recorded as shown in Figure 3.

Figure 3a presents the XPS spectra for the AlON layer on SiC after varying etching times. These spectra reveal the presence of several elements within the SiC/AlON interface, including Al, O, N, Si, and C. The detection of Ar in the survey spectra results from the use of Ar^+^ ion etching during the depth profiling process.

As the etching time increased, the elemental signals from the surface film gradually diminished, while signals from the substrate progressively intensified. During the initial etching stage, distinct peaks corresponding to elements such as Al, N, and O were observed, indicating that the surface primarily consisted of the AlON film. As the etching progressed (400–500 s), the signals of Si and C elements emerged and strengthened, signifying the approach towards the SiC substrate. In the later etching stages (700 s), Si and C signals became dominant, confirming that the SiC substrate had been reached. The atomic concentration profiles of the elements, obtained by integrating the areas of their characteristic peaks, are presented in Figure 3b. The carbon peak detected at the surface is likely attributed to adventitious carbon adsorbed onto the sample [30]. The elemental composition of the film, based on the XPS data, shows an approximate ratio of Al, O, and N of 5:1:4. The surface oxygen content is relatively high, which is attributed to oxidation during the sample transfer process. During the etching time between 300 and 500 s, the AlON film transitions to Al_2_O_3_. Around the 500 s etching time, the N element essentially disappears, and the Al and O elements dominate with a ratio close to 2:3, indicating that the surface in this region is primarily Al_2_O_3_. In the initial stage of ALD, there is a higher residual amount of O_2_ in the chamber and pipes, and O_2_ has high reactivity. Consequently, a thin Al_2_O_3_ layer initially forms on the surface of the substrate. During the etching time between 500 and 700 s, Al_2_O_3_ gradually transitions to the SiC substrate. Figure 3c presents the high-resolution core-level XPS spectra of various elements obtained at different etching times. The spectra of elements within the AlON film exhibit no significant shifts, suggesting that the deposited AlON layer has a relatively uniform distribution. However, at the interface, the Al 2p peak exhibits a large energy shift, which is strongly influenced by the chemical composition at the interface.

**Figure 4:**
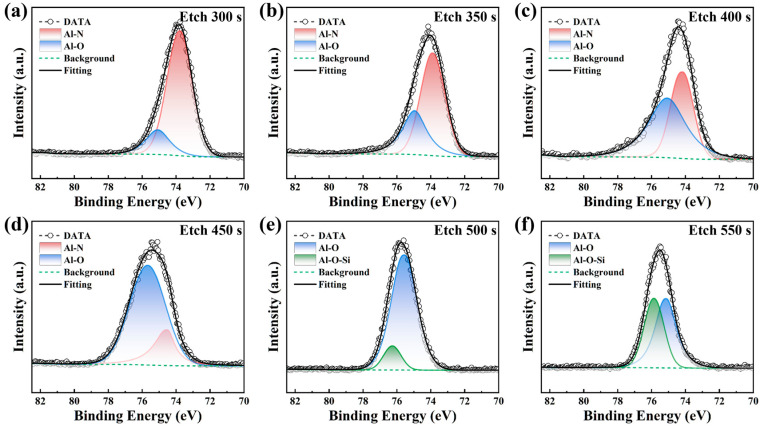
XPS spectra of Al 2p at various etching intervals and the corresponding peak fitting results: (**a**) 300 s; (**b**) 350 s; (**c**) 400 s; (**d**) 450 s; (**e**) 500 s; (**f**) 550 s.

The Al 2p spectrum of the AlON film as shown in Figure 4 exhibits three distinct peaks at 76 eV, 75.5 eV, and 73.5 eV, attributed to the Al-O-Si bond, Al-O bond, and Al-N bond, respectively [31,32,33]. As the etching time progresses, the Al 2p peak shifts to higher binding energies. After 300 s of etching, the Al 2p spectrum resolves into two distinct peaks corresponding to Al-O and Al-N. With prolonged etching, the Al-O peak intensity grows, whereas the Al-N peak intensity decreases. This suggests that nitrogen participated in the reaction later than oxygen during the initial stages of ALD, which can be attributed to the differing reactivity of the two elements. After 500 s of etching, the Al-N peak vanishes, and an Al-O-Si peak emerges in the Al 2p spectrum. As the etching time further increases, the proportion of Al-O-Si gradually increases. This suggests that in the region closer to the substrate, the Al and O sources react with the SiC, forming Al-O-Si bonds. Overall, the chemical composition at the AlON/SiC interface shows a gradual transition from Al-N and Al-O near the surface to Al-O and Al-O-Si closer to the substrate. This variation reflects the differences in the chemical composition of Al-based compounds at different interface depths and their evolution over the etching process. The study shows that, during annealing of AlN/SiC MOS, Al-O bonds and silicon suboxides at the AlN/4H-SiC interface can transform into more stable Al-N and Al-O-Si bonds [34]. The AlON films prepared exhibited a smooth transition in the interface without requiring annealing from Al-N/Al-O to Al-O-Si based on XPS analysis. This indicates that the interface of the AlON/SiC MOS prepared is inherently stable.

**Figure 5:**
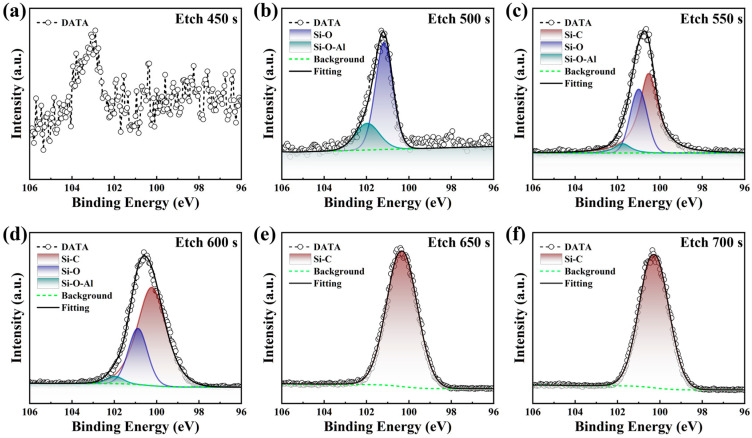
XPS spectra of Si 2p at different etching durations and their corresponding peak fitting results: (**a**) 450 s; (**b**) 500 s; (**c**) 550 s; (**d**) 600 s; (**e**) 650 s; (**f**) 700 s.

Figure 5 illustrates the Si 2p spectrum of the AlON film, which shows three prominent peaks at 100.1 eV, 101.1 eV, and 101.8 eV. These peaks are associated with the Si-C bond, Si-O bond, and Si-O-Al bond, respectively [18]. After 450 s of etching, the Si 2p core-level peak becomes undetectable.

With an etching time of 500 s, the Si 2p peak weakens in intensity and shifts to higher binding energies, indicating the formation of Si-O and Si-O-Al bonds. As the etching depth reduces, the Si-C peak intensity also decreases. After 600 s of etching, the Si 2p spectrum reveals the presence of Si-O and Si-O-Al bonds. The appearance of the Si-O bond points to limited oxidation of the SiC surface, likely resulting from the initial interaction between oxygen plasma and silicon during the early ALD process. The Si-O-Al bond indicates the role of oxygen atoms in forming the film network, linking the SiC substrate with the AlON layer [32]. During etching times between 650 and 700 s, the Si 2p XPS spectra exhibit a prominent peak at 100.7 eV, which is associated with the Si-C bond. This suggests that the surface in this etching range predominantly consists of the SiC substrate. Overall, as the etching depth increases, the chemical state of Si 2p gradually shifts from primarily Si-O-Al to Si-O and Si-C. This variation is attributed to the interaction of O plasma with the SiC surface during the ALD process, along with the underlying film growth mechanism [35].

The band offsets at the AlON/SiC heterojunction were assessed using XPS and valence band spectral analysis. The valence band offset (ΔE_V_) is typically assessed by measuring the energy difference between the peaks of the valence bands of AlON and SiC. Nonetheless, the interface’s chemical conditions can impact the electronic structure, leading to localized shifts in the energy levels near the junction. Consequently, conducting XPS measurements at the interface offers a more accurate representation of the electronic properties and associated energy shifts. At the AlON/SiC interface, the ΔE_V_ is calculated using the Kraut method, as shown below [36]:

(3) $$\Delta E_{V}=(E_{{\mathrm{Si}}_{2p}}^{\mathrm{SiC}}-E_{\mathrm{VBM}}^{\mathrm{SiC}})-(E_{{\mathrm{Si}}_{2p}}^{\frac{\mathrm{AlON}}{\mathrm{SiC}}}-E_{{\mathrm{Al}}_{2p}}^{\frac{\mathrm{AlON}}{\mathrm{SiC}}})-(E_{{\mathrm{Al}}_{2p}}^{\mathrm{AlON}}-E_{\mathrm{VBM}}^{\mathrm{AlON}})$$

In this equation, $E_{{\mathrm{Si}}_{2p}}^{\mathrm{SiC}}$ is the binding energy corresponding to the Si 2p peak in the SiC substrate, while $E_{{Si}_{2p}}^{\frac{AlON}{SiC}}$ is the binding energy of the Si 2p peak at the AlON/SiC interface. Furthermore, $E_{{\mathrm{Al}}_{2p}}^{\mathrm{AlON}}$ is the binding energy of the Al 2p peak within the AlON, whereas $E_{{\mathrm{Si}}_{2p}}^{\mathrm{SiC}}$ is the binding energy of the Al 2p peak at the AlON/SiC interface. Additionally, $E_{\mathrm{VBM}}^{\mathrm{SiC}}$ and $E_{\mathrm{VBM}}^{\mathrm{AlON}}$ represent the valence band maxima (V_BM_) for the SiC and the AlON. These values can be extracted by performing a linear extrapolation of the valence band spectra.

**Figure 6:**
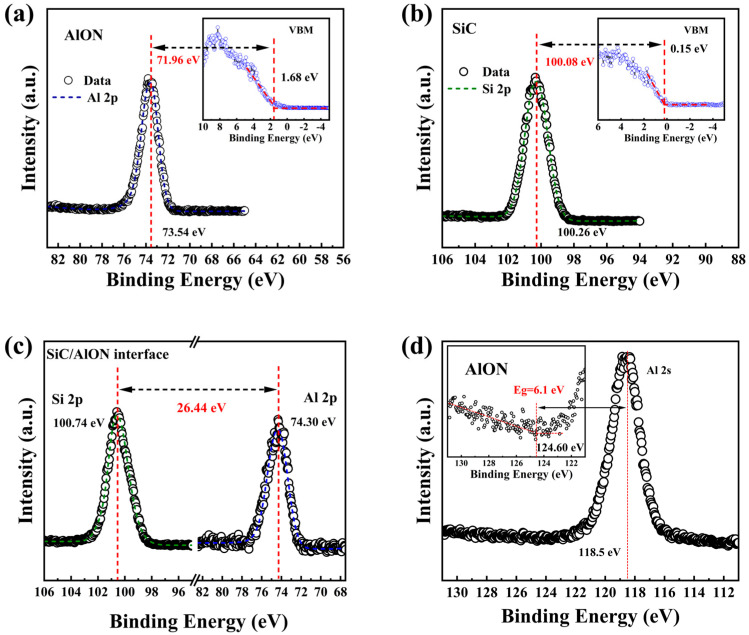
(**a**) Al 2p core-level and valence band analysis of AlON. (**b**) Si 2p core-level and valence band analysis of AlON. (**c**) Si 2p and Al 2p core-level spectra of the SiC/AlON interface. (**d**) Energy loss spectrum of the Al 2p peak of AlON.

Figure 6a displays the Al 2p core level for AlON, where a prominent peak appears at a binding energy of 71.96 eV. In comparison, the Si 2p core level for the SiC substrate, shown in Figure 6b, reveals a peak at a binding energy of 100.26 eV. By performing a linear extrapolation of the valence band data presented in Figure 6a,b, the V_BM_ values for AlON and SiC were determined to be 1.68 eV and 0.15 eV. The value of $E_{{\mathrm{Al}}_{2p}}^{\mathrm{AlON}}-E_{\mathrm{VBM}}^{\mathrm{AlON}}$ is calculated for 71.96 eV, while that of $E_{{\mathrm{Si}}_{2p}}^{\mathrm{SiC}}-E_{\mathrm{VBM}}^{\mathrm{SiC}}$ is 100.08 eV.

Figure 6c presents the core-level spectra at the interface of Si 2p and Al 2p, with corresponding binding energies of 100.74 eV and 75.30 eV. The value of $E_{{\mathrm{Si}}_{2p}}^{\frac{\mathrm{AlON}}{\mathrm{SiC}}}-E_{{\mathrm{Al}}_{2p}}^{\frac{\mathrm{AlON}}{\mathrm{SiC}}}$ is calculated to be 26.44 eV. Using the relationship outlined in Equation (3), the corresponding value is found to be 1.68 eV. The Al 2p binding energy in the AlON film and the Si 2p binding energy in the SiC substrate were chosen as the reference core levels for the gate dielectric and substrate, respectively. The maximum valence band energy levels for AlON and SiC were determined through linear extrapolation of their respective valence band spectra. The bandgap of the SiC substrate was taken as 3.26 eV based on theoretical data, while the bandgap of the AlON film was calculated to be 6.10 eV from the energy loss spectrum analysis of the Al 2s fine structure transition, shown in Figure 6d. This is consistent with the bandgap of AlON films obtained through linear extrapolation of the SE test curve in this work. The value of conduction band offset ($\Delta E_{C}$) can be derived using the energy band relationship:

(4) $$\Delta E_{C}=E_{g}(AlON)-\Delta E_{V}-E_{g}(SiC)$$

where $E_{g}\left(\mathrm{AlON}\right)$ is AlON’s bandgap and the extrapolated $E_{g}\left(\mathrm{AlON}\right)$ for AlON is taken as 6.10 eV, as shown in Figure 6d. While $E_{g}\left(\mathrm{SiC}\right)$ is for SiC, with the bandgap of SiC taken as 3.26 eV. The bandgap offset at the AlON/SiC interface is calculated to be 1.16 eV.

**Figure 7:**
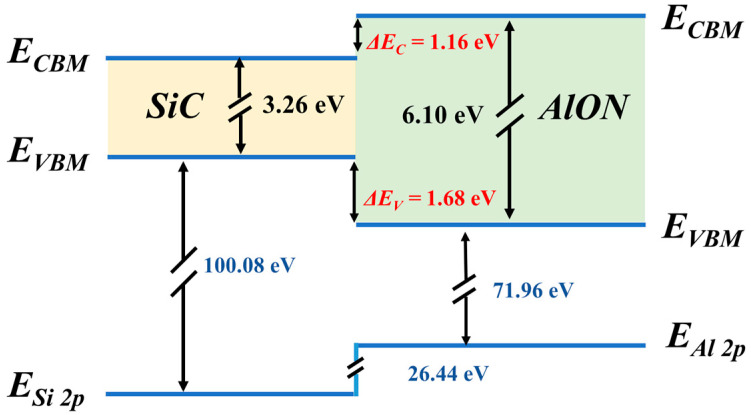
Band alignment of SiC and AlON.

The energy band alignment for the SiC/AlON heterostructure was computed, as shown in Figure 7. The findings reveal that the heterojunction formed between the AlON film and the SiC substrate is a Type I heterojunction, with a $\Delta E_{C}$ of 1.16 eV and a $\Delta E_{V}$ of 1.68 eV. Both band offsets exceed 1 eV, which is sufficient to effectively prevent charge carrier tunneling [37,38].

### 3.3. Electrical Properties of AlON/SiC MOS Capacitor

**Figure 8:**
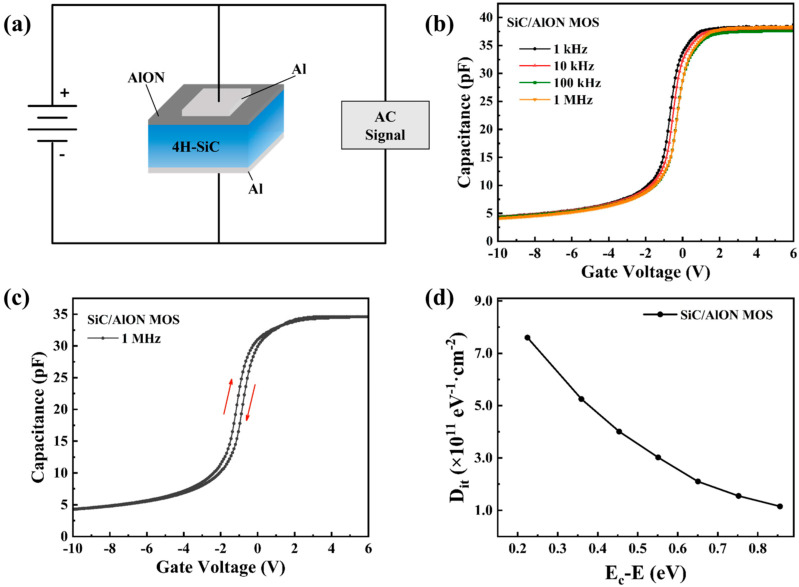
(**a**) Circuit configuration for C-V measurements. (**b**) C-V characteristics measured at various frequencies. (**c**) High-frequency hysteresis data for freshly prepared samples. (**d**) Interface defect density distribution as a function of energy.

The C-V characteristics of the SiC/AlON MOS capacitors were performed at 1 kHz, 10 kHz, 100 kHz, and 1 MHz using a probe station integrated with a semiconductor analyzer. The gate voltage varied between 10 V and 6 V. The equivalent circuit used for C-V measurement of the MOS capacitor is illustrated in Figure 8a. The C-V curve shown in Figure 8b exhibits the typical gate capacitance characteristics for SiC, with the applied gate voltage varying between −10 V and 6 V. This range corresponds sequentially to the deep depletion, depletion, and accumulation regions. The accumulation capacitance of the sample remains stable across various frequencies. The C-V curve exhibits a steep increase at low frequencies in the depletion region. As the test frequency increases, the slope of the curve decreases, which can be attributed to the capture and release of electrons by interface traps charges. These charges play a significant role in modulating the threshold voltage and influencing carrier mobility in MOS devices [39].

Figure 8c illustrates the C-V hysteresis characteristics of the fabricated SiC/AlON MOS capacitor, where the gate voltage is swept between depletion and accumulation, returning to depletion, at a frequency of 1 MHz under ambient conditions. A noticeable hysteresis is evident in the curves, typically attributed to the charging and discharging of near-interface traps (N_ITs_). During the reverse sweep, the C-V curve shifts by 0.32 V relative to the forward sweep. The density of near-interface traps, calculated by:

(5) $$N_{\mathrm{ITs}}=C_{\mathrm{OX}}\cdot \Delta V_{\mathrm{hy}}/qS$$

where C_OX_ represents the oxide layer capacitance, ΔV_hy_ is the difference between the V_FB_ under forward and reverse bias, q is the elementary charge, and S is the electrode area. N_ITs_ was determined to be 1.73 × 10^11^ cm^−2^. Quantitative data on the V_FB_ values are provided in Table 1. This table explicitly presents the V_FB_ measurements, highlighting the variations under different biasing conditions. These data are essential for a comprehensive understanding of the V_FB_ behavior and its impact on the device’s performance. A low N_ITs_ density helps reduce carrier trapping in the channel, thus improving the device’s stability under prolonged biasing [40].

The extraction of interface state density for 4H-SiC MOS capacitors is commonly performed by methods such as the high-frequency Terman method [41], the high-frequency quasi-static method [42,43], and the conductance method [44]. In particular, the high-frequency quasi-static method is advantageous for its relative simplicity and ability to quantitatively analyze interface states over a broad energy range (E_C_ − E_t_ = 0.2~0.8 eV). The tests were conducted using standard testing methods. At low or quasi-static frequencies, interface traps can effectively track changes in the voltage signal due to the slower electron capture process. However, at higher frequencies, the rate at which these traps capture and emit electrons becomes too slow to match the rapid fluctuations of the AC signal, reducing their impact on the capacitance response [45]. Additionally, the trap energy levels corresponding to D_it_ can be derived from the surface potential, which is related to the conversion of the gate voltage. The relationship between interface defect density and trap energy levels for the SiC/AlON MOS capacitor is shown in Figure 8d. These variations were quantitatively analyzed using the expression provided in [43]:

(6) $$D_{\mathrm{it}}=\frac{1}{\mathrm{Sq}}[{\left(\frac{1}{C_{\mathrm{LF}}}-\frac{1}{C_{\mathrm{OX}}}\right)}^{-1}-{\left(\frac{1}{C_{\mathrm{HF}}}-\frac{1}{C_{\mathrm{OX}}}\right)}^{-1}]$$

where C_LF_ and C_HF_ represent the capacitances at low and high frequencies, respectively, q is the elementary charge, and S is the electrode area. The D_it_ at an energy of E_C_—0.2 eV, near the conduction band, was found to be 7.6 × 10^11^ cm^−2^·eV^−1^. As shown in Figure 8d, the D_it_ versus E_C_−E curve exhibits a linear trend on a logarithmic scale, suggesting an exponential distribution of D_it_, with the defect density increasing toward the conduction band. The AlON film grown via PEALD demonstrates an excellent interface quality with SiC, primarily due to the ability of PEALD to achieve atomic-scale uniformity and prevent any misalignment or mismatch between layers.

**Figure 9:**
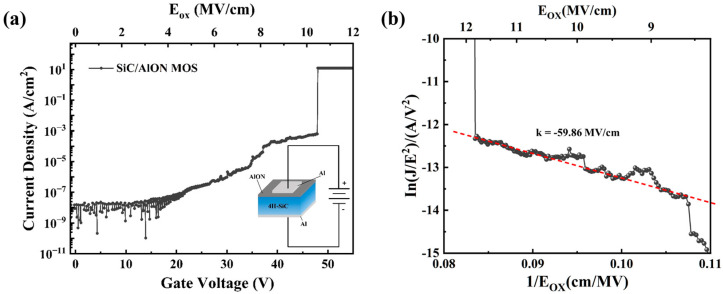
(**a**) J-E and J-V behavior of the SiC/AlON MOS capacitor. (**b**) FN J-E characteristics as a function of the applied electric field, along with a schematic illustrating the FN tunneling mechanism.

I-V measurements were performed on the SiC/AlON MOS capacitors, with the resulting curves of current density (J) as a function of gate voltage (V_G_) and electric field (E_OX_) shown in Figure 9a,b by J = I/S and $E_{\mathrm{OX}}\;=\;(V_{G}-V_{\mathrm{FB}})/t_{\mathrm{OX}}$ respectively. The leakage currents were assessed by incrementally increasing the gate voltage until the dielectric layer experienced irreversible breakdown. As depicted in Figure 9a shows a significant increase in leakage current at a gate voltage of 47.6 V, marking the onset of dielectric breakdown. This corresponds to a breakdown field of 10.4 MV/cm and a current density of 10^−8^ A/cm^2^.

The observed leakage current behavior is consistent with the characteristics of Fowler–Nordheim (F-N) tunneling. When a high positive voltage is applied to the metal (Al) relative to the semiconductor, the energy levels in the oxide layer near the metal are lowered, creating a triangular potential barrier. This enables electrons in the semiconductor to tunnel across the barrier and reach the metal. To further investigate the current electric field (J-E) behavior of the MOS structures, the F-N tunneling mechanism is applied. The current density due to F-N tunneling in the presence of strong electric fields can be described by the following equation [46]:

(7) $$J_{\mathrm{FN}}\;=\;AE^{2}\exp(-\frac{B}{E})$$

(8) $$A=\frac{q^{3}m_{e}}{8\pi hm_{\mathrm{ox}}\phi_{B}},\;B=\frac{8\pi {(2m_{\mathrm{ox}})}^{\frac{1}{2}}\phi_{B}^{\frac{3}{2}}}{3\mathrm{qh}}$$

where $m_{e}$ is the free electron mass, $m_{\mathrm{ox}}$ is the effective electron mass of AlON, and $\phi_{B}$ is the energy barrier at the AlON/SiC interface, h denotes the Planck constant, and q is the elementary charge. The relationship between $\ln({J/E}^{2})$ and 1/E_OX_ is shown in Figure 9b. A linear regression performed under high electric field conditions yields a B value of 59.86 MV/cm. The barrier height for AlON, calculated using a relative permittivity of 0.4, is found to be 1.24 eV, which aligns with the value obtained from the XPS analysis. The SiC/AlON interface shows a higher barrier height compared to the SiC/AlN interface [14,47], effectively preventing electron injection into the dielectric layer and consequently minimizing leakage current. Overall, the results from C-V and I-V measurements confirm that the SiC/AlON MOS capacitors feature a low interface state density, high breakdown field strength, and a significantly large barrier height, highlighting their excellent performance. Consequently, the fabricated AlON films possess exceptional properties that combine the advantages of Al_2_O_3_ and AlN, positioning AlON as promising gate dielectric candidates for SiC devices.

Table 2 provides an overview of recent studies on the properties of SiC/Al_2_O_3_, SiC/AlN, and SiC/AlON MOS structures. Compared to Al_2_O_3_/SiC MOS devices, the AlON/SiC MOS in this study achieved a higher breakdown field strength; however, there was a slight increase in the interface state density. In contrast, when compared to AlN/SiC MOS devices, the AlON/SiC MOS showed a slight decrease in breakdown field strength. Nevertheless, it demonstrated significant improvement in the interface quality, with the interface state density reduced by an order of magnitude. Additionally, when using a high NH_3_/O_2_ ratio as the nitrogen and oxygen source, the AlON/SiC MOS showed a slight decrease in breakdown field strength compared to those prepared with the N_2_/H_2_/H_2_O mixture at the same temperature, but the interface state density decreased by an order of magnitude. The SiC/AlON structure exhibits excellent breakdown performance and a low density of interface states.

Table 3 compares the interfacial properties of AlON/SiC with those of advanced high-k dielectrics such as HfO_2_ and ZrO_2_ in terms of D_it_ and breakdown field strength. Although HfO_2_ and ZrO_2_ possess relatively high dielectric constants, they generally exhibit a higher D_it_ at the SiC interface and a lower breakdown field strength, which to some extent limits their applicability in high-performance SiC devices. In contrast, the AlON films deposited by PEALD in this study demonstrate superior performance in both aspects. Specifically, AlON effectively reduces the D_it_ at the SiC interface, thereby improving the interfacial electrical characteristics, while maintaining a high breakdown strength. The combination of high dielectric performance and excellent interfacial quality highlights the significant advantages of AlON as a gate dielectric in SiC power devices.

## 4. Conclusions

AlON thin films are grown on SiC substrates using a high ratio of NH_3_ and O_2_ as nitrogen and oxygen sources through PEALD technology firstly in this study, with a comprehensive evaluation of both material properties and electrical performance. The AlON films exhibited uniform thickness with a minimal relative error of only 0.14%, a high refractive index of 1.90, and an RMS surface roughness below 1 nm, showcasing the precision of the PEALD technique. XPS depth profiling indicated a well-defined interface between AlON and SiC, with a smooth transition from Al-N and Al-O near the surface to Al-O and Al-O-Si at the interface with the substrate, ensuring robust bonding. Electrical measurements revealed that the SiC/AlON MOS capacitors had a ΔE_C_ of 1.16 eV and a ΔE_V_ of 1.68 eV, resulting in Type I band alignment that effectively prevents carrier tunneling. The D_it_ was 7.6 × 10^11^ cm^−2^·eV^−1^, and the N_ITs_ was 1.73 × 10^11^ cm^−2^, both of which were low, indicating a high-quality interface. Additionally, the breakdown electric field of the SiC/AlON MOS capacitor reached 10.4 MV/cm, demonstrating its high dielectric strength. These findings indicate that AlON holds great potential as a gate dielectric for SiC MOS devices. It provides enhanced interface quality, minimal defect density, and exceptional breakdown strength, thereby boosting the performance and reliability of high-voltage, high-power SiC devices.

## Figures and Tables

**Table 1:** Quantitative data of V_FB_ value.

Ideal V_FB_	Forward Bisa V_FB_	Reverse Bisa V_FB_	ΔV_hy_
0.15 V	−0.48 V	−0.8 V	0.32 V

**Table 2:** Properties of Al_2_O_3_, AlN, and AlON films grown on 4H-SiC via different deposition methods, as documented in the literature.

Sample	Method	D_it_ at E_C_—0.2 eV	E_BR_	Reference	Time
Al/Al_2_O_3_/SiC	Hot plate at 200 °C	1.2 × 10^10^ eV^−1^·cm^−2^	5.2 MV/cm	[9]	2018
Ti/Al/AlN/SiC	PEALD (TMA and NH_3_)	1.85 × 10^13^ eV^−1^·cm^−2^	—	[14]	2024
Al/AlN/SiC	PEALD at 200 °C (TMA and N_2_/H_2_)	4.26 × 10^12^ eV^−1^·cm^−2^	10.9 MV/cm	[13]	2020
Al/AlON/SiC	PEALD at 200 °C (TMA and N_2_/H_2_/H_2_O)	8.93 × 10^12^ eV^−1^·cm^−2^	11.4 MV/cm	[13]	2020
Al/AlON/SiC	PEALD at 200 °C (TMA and NH_3_/O_2_)	7.6 × 10^11^ eV^−1^·cm^−2^	10.4 MV/cm	This work	2025

**Table 3:** Properties of ZrO_2_ and HfO_2_ films grown on 4H-SiC via different deposition methods, as documented in the literature compared with AlON film.

Sample	Method	D_it_ at E_C_—0.2 eV	E_BR_	Reference	Time
ZrO_2_/SiC	TALD at 270 °C (TDMAZ)	1.58 × 10^12^ eV^−1^·cm^−2^	3.76 MV/cm	[48]	2025
ZrO_2_/SiC	TALD at 270 °C (TDMAZ and O_2_)	9.5 × 10^12^ eV^−1^·cm^−2^	5.9 MV/cm	[49]	2024
HfO_2_/SiC	TALD at 250 °C (TEMAH and H_2_O)	2.1 × 10^13^ eV^−1^·cm^−2^	—	[50]	2018
HfO_2_/SiC	PEALD (TEMAH and O_2_)	—	5.8 MV/cm	[51]	2019
Al/AlON/SiC	PEALD at 200 °C (TMA and NH_3_/O_2_)	7.6 × 10^11^ eV^−1^·cm^−2^	10.4 MV/cm	This work	2025

## Data Availability

The original contributions presented in this study are included in the article. Further inquiries can be directed to the corresponding author.

## Abbreviations

SiC	Silicon Carbide
Si	Silicon
PEALD	Plasma-Enhanced Atomic Layer Deposition
RF	Radio Frequency
AlN	Aluminum Nitride
Al_2_O_3_	Aluminum Oxide
AlON	Aluminum Oxynitride
TMA	Trimethylaluminum
BOE	Buffered Oxide Etch
AFM	Atomic Force Microscopy
XRD	X-ray Diffraction
XPS	X-ray Photoelectron Spectroscopy
SE	Ellipsometry
N_ITs_	Near-Interface Traps
N_eff_	Effective Fixed Charge Density
D_it_	Interface State Density
I-V	Current–Voltage
C-V	Capacitance–Voltage
FN	Fowler–Nordheim
ΔE_V_	Valence Band Offset
ΔE_C_	Conduction Band Offset
V_BM_	Valence Band Maxima
J	Current Density
J-E	Current Electric Field
V_G_	Gate Voltage
